# A Look-Up Table Assisted BiLSTM Neural Network Based Digital Predistorter for Wireless Communication Infrastructure

**DOI:** 10.3390/s25134099

**Published:** 2025-06-30

**Authors:** Reem Al Najjar, Oualid Hammi

**Affiliations:** Department of Electrical Engineering, College of Engineering, American University of Sharjah, Sharjah P.O. Box 26666, United Arab Emirates

**Keywords:** 5G communications, bidirectional long-short term memory, neural networks, nonlinear distortions, power amplifiers, predistortion

## Abstract

Neural networks are increasingly attractive for digital predistortion applications due to their demonstrated superior performance. This is mainly attributed to their ability to capture the intrinsic traits of nonlinear systems. This paper presents a novel hybrid predistorter labeled as the look-up table assisted bidirectional long-short term memory (BiLSTM) neural network (LUT-A-BiNN) that combines a neural network cascaded with a look-up table in a manner that both sub-models complement each other. The main motivation in using this two-box arrangement is to eliminate the highly nonlinear static distortions of the PA with the look-up table, allowing the neural network to focus on the compensation of the dynamic distortions. The proposed predistorter is experimentally validated using 5G test signals. The results demonstrate the ability of the proposed predistorter to achieve a 5 dB enhancement in the adjacent channel leakage ratio when compared to its single-box counterpart (BiLSTM neural network predistorter) while maintaining the signal-agnostic performance of the BiLSTM predistorter.

## 1. Introduction

Networking, data transmission, and radiofrequency electronics are several key factors involved in the design of wireless infrastructure for 5G communication systems. The operation of the transmitters’ RF front-end, especially the RF power amplifier (PA), is subject to very strict constraints stemming from 5G systems requirements. This is mainly determined in terms of transmit signal quality quantified through metrics such as the adjacent channel leakage ratio (ACLR) and the error vector magnitude (EVM) [1]. The enhancement of high data transmission efficiency can be achieved by ensuring that the transmitted signals are free of distortions. A straightforward approach would be to design linear power amplifiers and operate them at enough back-off to ensure linear amplification. However, this will have detrimental effects on the amplifier’s power efficiency which has a direct impact on the energy utilization in base stations and IoT gateways. Therefore, it is essential to operate power amplifiers in their nonlinear region while generating controllable distortions that can be eliminated through system level linearization techniques [1,2,3,4].

Radio frequency power amplifiers play a crucial part in communication networks infrastructure. Emerging wireless communication standards continuously set more challenging constraints on the performance of PAs and more specifically their expected linearity. Enhancing the linearity of power amplifiers to standard compliant levels has been an active research area in which new challenges arise with the adoption of each and every communication standard. Many approaches have been proposed to linearize power amplifiers with a clear emphasis on digital predistortion techniques, as they often result in the best trade-off in terms of implementation complexity and linearization performance [1]. A digital predistorter (DPD) is a nonlinear function implemented in the digital domain that will cause a nonlinearity complementary to that of the power amplifier in a manner that the cascade made of the DPD and PA nonlinear systems behaves as a linear amplification system.

Over the last few years, machine learning techniques have been widely used for the enhancement of base stations efficiency in wireless communication systems through predistortion applications [5,6,7,8,9,10,11,12,13,14,15,16,17,18,19]. Indeed, several types of neural networks, such as conventional feedforward neural networks [8,9], and their augmented versions [10], convolution neural networks (CNNs) [11,12,13,14], recurrent neural networks (RNNs) [15,16,17], and vector decomposition based neural networks [18,19], have been proposed as digital predistortion functions. Among these, convolution neural networks and long-short term memory (LSTM) neural networks have demonstrated highly attractive features with their ability to capture intrinsic behavior of the power amplifier and hence allow for maintaining the predistorter performance over a wide range of operating conditions [13,14,15]. Recently reported results demonstrated the ability of the bidirectional LSTM (BiLSTM)-based neural networks to achieve signal agnostic performance [15].

Two-box based predistorters have been widely used for analytically defined functions. Wiener [20,21,22], Hammerstein [21,22,23], and twin-nonlinear two-box models [24], as well as their augmented versions [1,2,23], are examples for such implementations. In these cases, the identification of the two predistortion functions is typically conducted from a single measurement through a de-embedding process [20]. Moreover, two box-models can be used in two-stage predistortion systems where each of the predistortion functions is identified sequentially [25,26,27]. Two-box predistorters have clearly shown superior performance when compared to their single-box counterpart. This includes achieving better adjacent channel leakage ratio at the output of the linearized amplifier, and/or reducing the predistorter’s overall complexity while maintaining its performance.

This work aims at enhancing the trade-off between complexity and performance for neural networks based predistortion using a two-box configuration. For this purpose, a two-box structure labeled look-up table assisted BiLSTM neural network (LUT-A-BiNN) predistorter is proposed. The resulting predistorter is made of the cascade of a neural network followed by a memoryless look-up table function. The main motivation is to compensate for the highly nonlinear memoryless distortions with the look-up table sub-function and allow the neural network to focus on the compensation of the residual distortions. The proposed predistorter is experimentally validated using various 5G new radio (NR) test signals. The results show that the proposed two-box arrangement allows for enhancing the ACLR performance of the standalone BiLSTM predistorter by approximately 5 dB while maintaining its signal’s bandwidth agnostic performance.

In Section 2, the proposed LUT-A-BiNN predistorter is presented along with its identification process. The experimental validation and performance benchmarking are detailed in Section 3, and the conclusions are summarized in Section 4.

## 2. Limitation of Single Box Models

Single box predistorters are used either as standalone functions or as part of a multi-box predistorter. Volterra models were initially created as Taylor series-based polynomials to model memory effects or more generally dynamic nonlinear systems [1]. However, in order to deal with substantially nonlinear behavior, Volterra series will have a very large number of coefficients as the nonlinearity order and memory depth of the system increase. There are numerous ways to reduce the size of the Volterra model through various pruning techniques [28,29,30,31]. One of its offspring is the memory polynomial (MP) model that can be used to approximate and reduce the number of DPD coefficients from the Volterra series by keeping only its diagonal terms. This version of the Volterra series has been preferred in many applications due to its simplicity of implementation and acceptable performance. MPs can accurately mimic the complex nonlinear properties of PAs due to their capacity to consider past input signals [1]. Although using a memory polynomial as a DPD yields good performance in the PA’s linearization, it can also rapidly increase the DPD’s complexity. As the memory depth increases, the MP model introduces many more coefficients. As a result, both the computing load desired to calculate the predistorted signal and the model’s complexity increase. Limited generality is another drawback brought about by the use of MP-based DPDs. In fact, PA behavior can frequently change due to variations in the signal bandwidth, power, or operating frequency. Such changes will cause a mismatch between the new PA behavior and the MP DPD, resulting in a loss of linearization performance. In such conditions, a closed loop scheme in which the MP DPD function is continuously updated is needed in order to maintain the linearization performance when the PA behavior changes.

Another type of single box predistorter is one that uses artificial intelligence (AI) and machine learning techniques. Neural networks (NNs) have recently witnessed wide adoption for predistortion applications and are useful in overcoming the difficulties presented by the high order modulation schemes used in 5G systems. This is due to the NNs capacity to capture complex nonlinearities present in PAs and their robustness to a variety of operating conditions [13,14,15]. NNs provide higher flexibility and generalization when compared to analytically defined models such as MPs or look-up tables, providing improved performance across a wider range of operational situations in communications systems. However, this generalization ability comes at the cost of higher complexity due to the number of coefficients in such models (weights and biases) often being much larger than that of analytically defined models. The large number of parameters is also accompanied by a significant training overhead. Thus, reducing the number of parameters will reduce the training overhead as well as the computational complexity needed to predict the output of the predistorter in the inference mode of the neural network.

Two-box models have been proposed for the behavioral modeling and predistortion of power amplifiers in the presence of memory effects. The main motivation is to have a sub-model dedicated to the highly nonlinear memoryless distortions and another sub-model that is tailored to the mildly nonlinear memory effects. For example, for mild memory effects, Wiener and Hammerstein models have been used. These have been enhanced by incorporating weakly nonlinear memory effects in what are known as the augmented Wiener and augmented Hammerstein models. More nonlinear memory effects can be modeled using twin-nonlinear two-box models and their variants. In general, two-box models were found to lead to better performance than their single-box counterparts while also having less complexity. Therefore, in this paper, an attempt to devise a two-box model structure using neural networks is proposed. This is achieved by cascading a neural network with a memoryless look-up table. The proposed structure is expected to enhance the tradeoff between the model performance and its complexity when compared to the standalone neural network single-box predistorter.

## 3. Proposed LUT-A-BiNN Predistorter Architecture

### 3.1. BiLSTM Neural Networks Architecture

**Figure 1:**
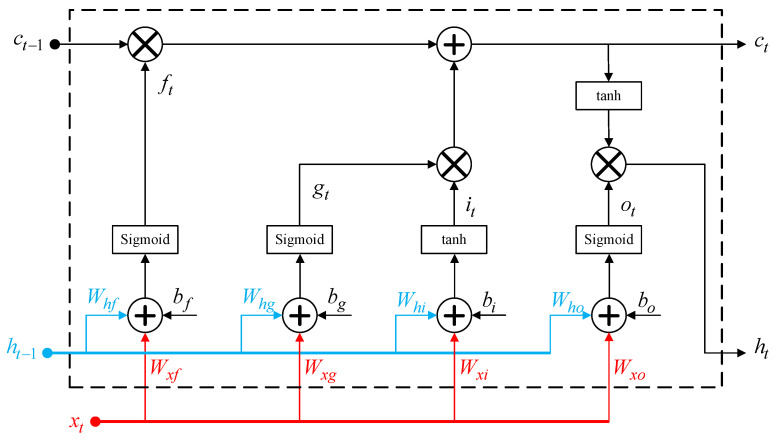
Architecture of the LSTM cell.

The BiLSTM neural networks represent an extension of the LSTM structure. As seen in Figure 1, the long short-term memory (LSTM) cell shows a sophisticated gating system that consists of a forget gate, an input gate, and an output gate [32]. The LSTM cell is fed by the input signal as well as the outputs of the preceding cell. This design facilitates selective long-term storage and retrieval of information by LSTMs, which helps in capturing intricate temporal relationships in data. The primary advantage of LSTMs cells is their ability to learn and retain meaningful context across a variety of time steps, which is crucial for applications such as the modeling of memory effects in nonlinear power amplifiers.

The following formulas can be used to determine the LSTM cell output $\left(c_{t}\right)$ and hidden state $\left(h_{t}\right)$ as a function of the input signal $\left(x_{t}\right)$ and the outputs of the preceding cell $\left(c_{t-1}\;\mathrm{and}\;h_{t-1}\right)$:

(1) $$c_{t}=\left(f_{t}⊗c_{t-1}+i_{t}⊗g_{t}\right)$$

(2) $$h_{t}=\left(o_{t}⊗\tanh\left(c_{t}\right)\right)$$

where ⊗ refers to the convolution operator. $f_{t}$, $g_{t}$, $i_{t}$, and $o_{t}$ are the internal parameters of the LSTM cell given by

(3) $$f_{t}=\sigma \left(W_{xf}x_{t}+W_{hf}h_{t-1}+b_{f}\right)$$

(4) $$g_{t}=\sigma \left(W_{xg}x_{t}+W_{hg}h_{t-1}+b_{g}\right)$$

(5) $$i_{t}=\tanh\left(W_{xi}x_{t}+W_{hi}h_{t-1}+b_{i}\right)$$

(6) $$o_{t}=\sigma \left(W_{xo}x_{t}+W_{ho}h_{t-1}+b_{o}\right)$$

The weights connected to the forget gate are $W_{xf}$ and $W_{hf}$, while the weights associated with the input gate are $W_{xg}$, $W_{hg}$, $W_{xi}$, and $W_{hi}$. The weights associated with the output gate are $W_{xo}$ and $W_{ho}$. $b_{g}$ and $b_{i}$ are the biases associated with the input gate. $b_{f}$ and $b_{o}$ are the biases values for the forget gate and the output layer, respectively.

The forget gate’s operation is described by Equation (1); the sigmoid function controls the gate’s output and determines whether to keep or discard historical data. The output $f_{t}$ of the forget gate is convoluted with the output $c_{t-1}$ of the preceding LSTM cell. The resulting signal is combined additively with the output of the input gate to generate the cell’s output signal $c_{t}$. The output of the input gate is obtained by convoluting the signals $g_{t}$ and $i_{t}$ generated within the input gate. These signals are obtained by applying to the cell’s inputs $x_{t}$ and $h_{t-1}$, a sigmoid and a tanh activation function, respectively. In the output gate, the cell’s output signal $c_{t}$ and the cell’s input signals ($x_{t}$ and $h_{t-1}$) are convoluted after being transformed using a tanh and sigmoid activation functions, respectively. This results in the cell’s output signal $h_{t}$.

A significant advancement in LSTM architecture is the bidirectional long short-term memory networks, which are designed specifically to utilize data from sequential data’s past and future time steps. By using two distinct LSTM layers, one of which processes input in a forward sequence and the other in a reverse sequence, BiLSTMs improve the model’s understanding of temporal links by including contextual features from both sides [33]. This bidirectional method improves the performance of BiLSTMs in many applications by allowing them to effectively capture context and long-range correlations in data sequences. A BiLSTM-based DPD model was presented in [16]. It consisted of a 300-neuron BiLSTM layer, which is followed by two fully connected layers with 150 and 200 neurons, respectively. The BiLSTM-based architecture performs better than the unidirectional LSTM-based architecture at the expense of additional memory state coefficients.

### 3.2. LUT-Assisted BiLSTM Neural Network Architecture

**Figure 2:**
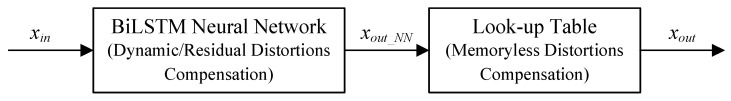
Simplified block diagram of the proposed LUT-A-BiNN DPD.

Two-box DPD structures have been proposed with the aim of enhancing the DPD performance and/or achieving a noticeable complexity reduction. Most of the two-box DPD structures attempt to split the dynamic nonlinear behavior of the power amplifier being linearized into a memoryless nonlinear behavior and quasi-linear memory effects. This applies to the Wiener and Hammerstein-based predistorters, as well as twin-nonlinear two-box DPDs. In this work, a look-up table-assisted BiLSTM neural network predistorter is proposed. While the arrangement of the two-box structure can be flexible in the sense that the look-up table can be used upstream or downstream of the neural network, the structure of Figure 2 has been adopted. This is mainly due to its suitability for a two-step identification, as described below. The main motivation is to use the look-up table structure to perform coarse linearization of the power amplifier behavior and compensate for the memoryless strongly nonlinear distortions. Therefore, focusing the neural network modeling capabilities on the more intricate memory effects as well as the residual distortions not accounted for in the look-up table. The identification of the two-sub functions of the LUT-A-BiNN predistorter can be performed in two different ways. One approach, using a single data acquisition, is similar to what is commonly used in two-box predistorters, i.e., the memoryless nonlinear function (implemented in the LUT) is identified first, and the data is de-embedded to the input and output planes of the neural network sub-function, which is subsequently trained. A second approach consists of identifying the LUT sub-function and applying it to linearize the DUT. The input of the LUT and the output of the coarsely linearized DUT are then used to train the neural network sub-function.

**Figure 3:**
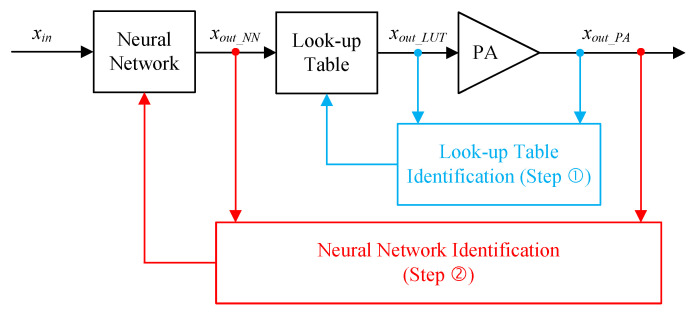
Simplified block diagram of the identification process adopted for the proposed LUT-A-BiNN DPD.

In this work, the second approach was adopted for the identification of the LUT-A-BiNN DPD function and its experimental validation. The identification process of the proposed LUT-A-BiNN DPD is depicted in Figure 3. The DUT is first characterized and its baseband input and output waveforms $x_{out\_LUT}$ and $x_{out\_PA}$ are used to derive the LUT based memoryless predistorter. This first step uses the inner characterization loop. The LUT predistorter is then applied to linearize the DUT, and the input and output baseband waveforms of the cascade made of the LUT DPD and the DUT (that is $x_{out\_NN}$ and $x_{out\_PA}$) are used to train the NN-based predistortion function. This second step uses the outer characterization loop. The selection of this sequential identification approach is mainly due to the demonstrated ability to extend the correction bandwidth of an experimental setup [26,27]. In our case, this was needed due to the limited bandwidth capability of the experimental setup (125 MHz), which is much lower than the optimal bandwidth (200 MHz) required for the used 40 MHz test signal.

## 4. Experimental Validation and Performance Benchmarking

### 4.1. Experimental Setup

**Figure 4:**
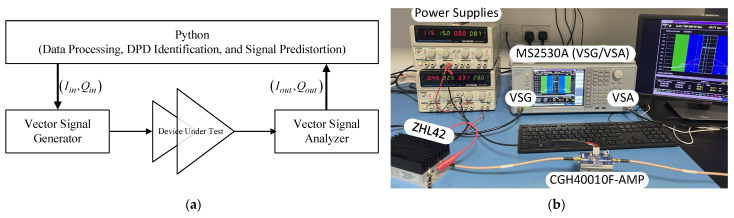
Experimental setup. (**a**) Block diagram, (**b**) photograph.

The experimental setup used in this work consists of the MS2830A (from Anritsu, Kanagawa, Japan) which includes a vector signal generator and a vector signal analyzer in the same chassis. The instrument has a maximum signal generation and analysis bandwidths of 125 MHz. Hence, limiting the bandwidth of the signal being used in the experimental validation to 40 MHz. The device under test is made of a two-stage power amplification system. The main amplifier is the CGH40010F-AMP evaluation board from Wolfspeed, Durham, NC, USA. The ZHL42 (from Minicircuits, Brooklyn, NY, USA) was used to drive the main amplifier. All digital signal processing, including identifying both DPD functions and predistorting the input signal, were implemented in Python 3.10. A block diagram of the experimental setup is shown in Figure 4.

In the experimental validation presented in this work, several 5G NR test signals were used. These signals have bandwidths varying from 10 MHz to 40 MHz. The bandwidths, sampling rates, as well as the PAPR of these test signals are summarized in Table 1.

### 4.2. BiLSTM DPD

**Figure 5:**
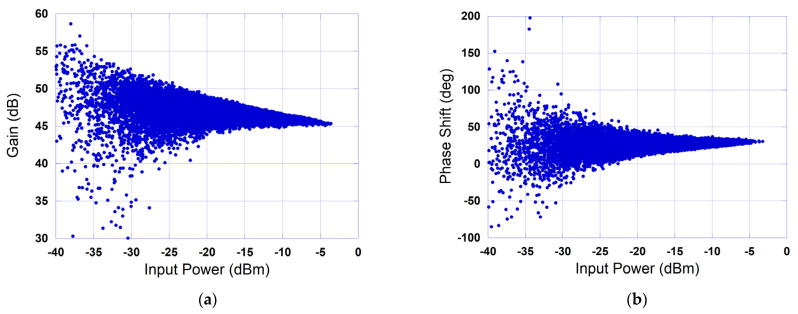
Measured characteristics of the device under test. (**a**) AM/AM characteristic, (**b**) AM/PM characteristic.

Several standalone neural networks have been applied for the linearization of the device under test through digital predistortion. These include the real-valued feedforward neural network [9], the convolutional neural network [11], the vanilla and the stacked LSTM neural networks [16], and the BiLSTM neural network [16]. This experimental benchmarking was carried out using a 4-carrier 40 MHz 5G NR test signal sampled at 153.6 Msps. The signal had a peak to average power ratio (PAPR) of 10.7 dB. The characterization of the DUT as well as its linearization were performed at an average power back-off that is equal to the signal’s PAPR. The AM/AM and AM/PM characteristics of the DUT measured under these conditions are reported in Figure 5. These characteristics clearly show the strongly nonlinear behavior and the memory effects of the device under test.

The linearization performances of the various neural network-based DPDs are summarized in Table 2. According to these results, it appears that the BiLSTM leads to better ACLR than the other models, and therefore it was selected as the neural network structure in the proposed two-box DPD. Moreover, the BiLSTM has recently been found to achieve signal-agnostic performance by being resilient to signal characteristics [15]. Hence, making it an attractive option for NN-based digital predistorters. The superiority of BiLSTM DPDs can be attributed to the fact that LSTM cells provide a sophisticated gating system that consists of memory cells and particular input, output, and forget gates. This design facilitates selective long-term storage and retrieval of information by LSTMs, which helps them capture intricate temporal relationships in data. The primary advantage of LSTMs is their ability to learn and retain meaningful context across a variety of time steps.

**Figure 6:**
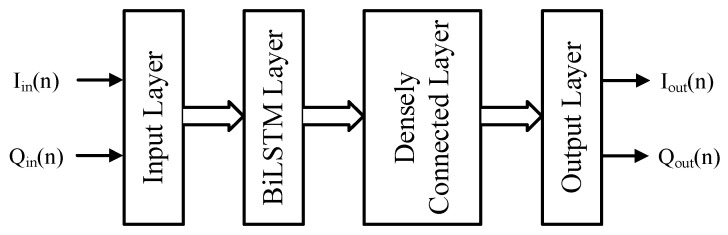
Simplified block diagram of the BiLSTM based neural network.

The BiLSTM neural networks used in this work are made of two layers in addition to the input and output layers as depicted in Figure 6. The input layer shapes the input data to feed the BiLSTM layer with the vector made of the present sample as well as *K* preceding samples (where *K* depends on the memory depth of the DUT). The BiLSTM layer is made of a total of $2N_{LSTM}$ LSTM cells for both forward and backward propagations. The outputs of the BiLSTM cells are fed into a densely connected neural network layer that feeds the 2-neurons of the output layer. The number of neurons in the densely connected layer is $N_{DNN}$. For the considered structure of Figure 6, the total number of parameters $\left(P\right)$, including weights and biases, of the neural network block is given by

(7) $$P=8{\left(N_{LSTM}\right)}^{2}+N_{DNN}\left(3+2N_{LSTM}\right)+24N_{LSTM}+2,$$

### 4.3. Performance Validation of the LUT-Assisted BiLSTM DPD

**Figure 7:**
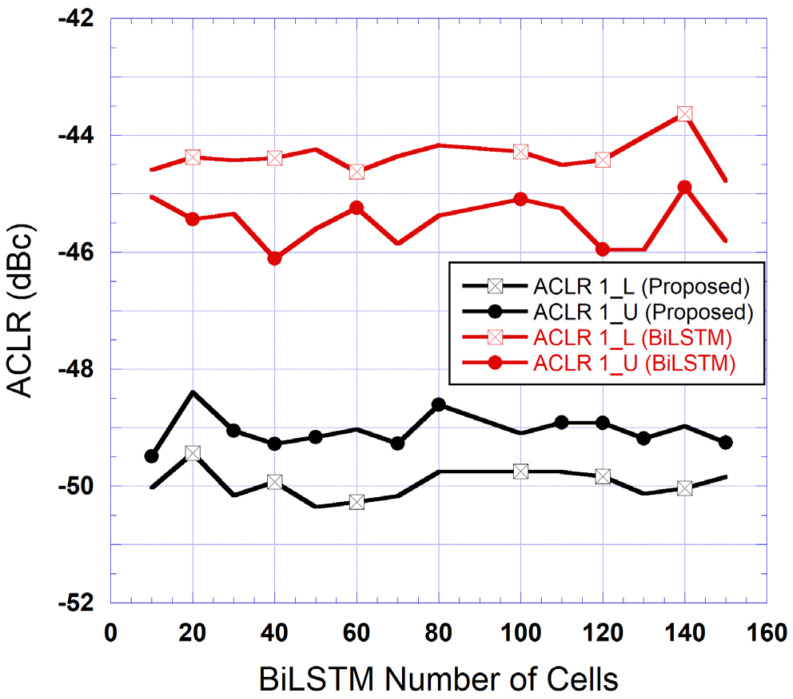
Measured ACLR at the output of the linearized DUT for various numbers of LSTM cells.

The first step of the experimental validation consisted of assessing the impact of the number of parameters of the BiLSTM sub-block on the performance of the proposed two-box DPD. Based on Equation (7), it appears that the impact of $N_{LSTM}$ on the total number of the BiLSTM parameters $\left(P\right)$ is much more significant than that of $N_{DNN}$. For this work, $N_{LSTM}$ was varied from 10 to 150 and the $N_{DNN}$ was varied between 80 and 200 with the aim of minimizing the overall number of parameters in the model while maintaining an ACLR of approximately −50dBc. The target ACLR value was selected to allow for a margin with respect to the 5G minimal requirement of −45dBc. From this study, it was found that for $N_{DNN}=100$, the targeted ACLR could be met with the lowest complexity. Figure 7 presents the measured ACLR at the output of the device under test as a function of $N_{LSTM}$ for the proposed DPD and for the standalone BiLSTM DPD. These results show that the proposed DPD consistently outperforms the standalone BiLSTM DPD by approximately 5 dB. Furthermore, the proposed DPD achieves the desired performance for $N_{LSTM}=10$, while the standalone BiLSTM DPD is unable to reach this performance even for much larger values of $N_{LSTM}$.

**Figure 8:**
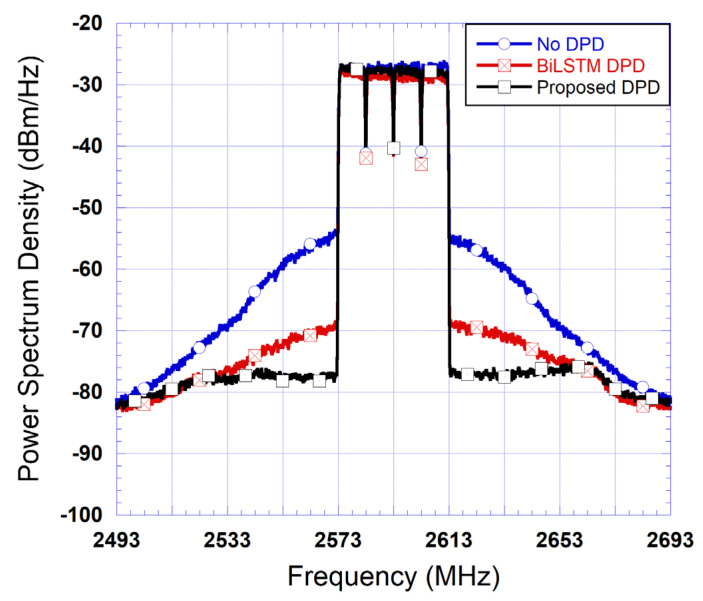
Measured spectra at the output of the DUT.

The measured spectra at the output of the DUT for the proposed DPD and the standalone BiLSTM DPD are reported in Figure 8. In this figure, both DPD are derived for $N_{DNN}=100$ and $N_{LSTM}=10$. This figure shows that the proposed DPD was able to linearize quasi-perfectly the DUT. These results also show a noticeable enhancement in performance when compared to the standalone BiLSTM predistorter.

Table 3 reports the measured ACLR at the output of the DUT for various DPDs including the LUT DPD, the memory polynomial (MP) DPD, the standalone BiLSTM DPD, and the proposed DPD. In this table, ACLR1 refers to the ACLR in the adjacent channels, while ACLR2 refers to the ACLR in the alternate adjacent channel. These results show that the LUT DPD performance is limited as expected due to the presence of strong memory effects in the DUT behavior. The use of the proposed DPD structure further enhances the linearization performance of the LUT DPD by an additional 10 dB. Most importantly, a 5 dB reduction in ACLR1 is obtained when compared to the standalone BiLSTM DPD without significant complexity overhead. This is consistent with the spectra depicted in Figure 8. Furthermore, the proposed BiLSTM-based two-box DPD structure leads to results comparable to those achieved with the MP DPD. The MP DPD performance is often considered as a reference. However, the performance of MP DPDs is quite sensitive to the operating condition of the PA in the sense that a new set of coefficients of the MP DPD needs to be computed whenever the average power of the bandwidth of the signal changes. However, neural network models such as CNN and BiLSTM were found to be agnostic to some variations in the operating conditions. For example, in [15], a BiLSTM DPD able to linearize a PA driven by signals of various bandwidths and power levels was reported. Furthermore, convolutional neural network-based DPDs were reported for the case of mismatched transmitters [13], and for the cases of power and temperature variations [14].

Accordingly, a very important advantage of neural networks when compared to analytically defined models is their ability to be generalized for a wide range of operating conditions. In prior work, the standalone BiLSTM DPD was found to be signal agnostic [15]. Hence, the resilience of the proposed DPD to changes in the PA drive signal bandwidth was assessed. For this purpose, 5G NR signals of various bandwidths were generated. Three types of DPDs were derived. First, The DUT was characterized using each of these signals, and the proposed was DPD derived. This type of DPD is referred to in Table 4 as “Proposed Matched”. This is because such DPDs are built using the proposed LUT-assisted BiLSTM structure and the signal used in the characterization step is the same as the one used in the linearization step. In the second test, one single DPD using the proposed LUT assisted BiLSTM structure was derived using the 40 MHz 4-carrier signal. This DPD was later applied to linearize the DUT while the driving signal was varied. In Table 4, this second DPD is referred to as “Proposed Mismatched” since it used the proposed LUT-assisted BiLSTM DPD while the signals used during the DPD identification step and the DUT linearization step are different. During the third test, the process followed was identical to that of the second test except that the DPD was built using a standalone BiLSTM structure. Therefore, the results corresponding to this case are labeled as “BiLSTM Mismatched” in Table 4.

**Figure 9:**
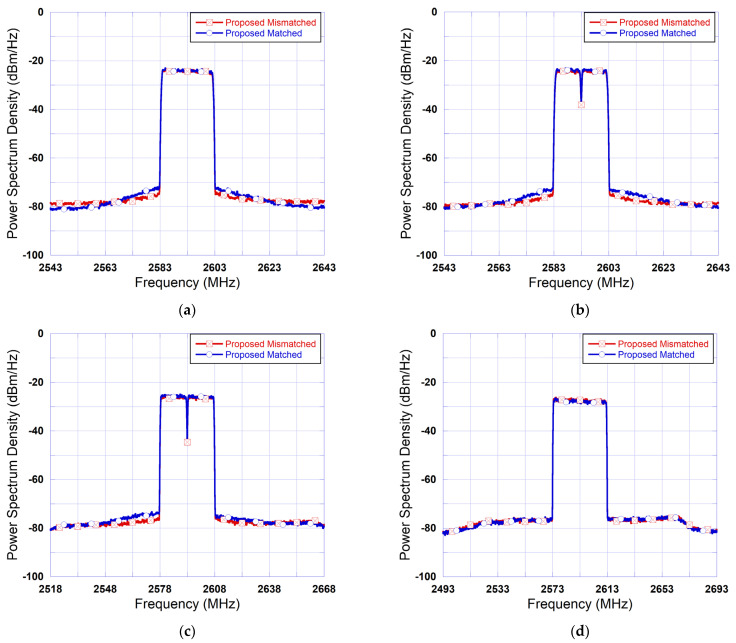
Measured spectra at the output of the linearized DUT using proposed matched and mismatched DPD. (**a**) 20 MHz 1-carrier signal, (**b**) 20 MHz 2-carriers signal, (**c**) 30 MHz 2-carriers signal, (**d**) 40 MHz signal 1-carrier signal.

Thus, in the first test, a DPD was derived for each test signal. Conversely, in the second test only one DPD was trained, and it was used to linearize the DUT driven by six different signals. The same applies to the third test. The results reported in Table 4 summarize the performance of the LUT-assisted BiLSTM DPD and the standalone BiLSTM DPD when applied to linearize the DUT driven by a set of unseen signals with various bandwidths. The results reported in this table show that the proposed mismatched DPD achieves comparable performance to that of the proposed matched DPD. This confirms the resilience of the proposed architecture to signal bandwidths within the tested range. Moreover, comparing the proposed mismatched DPD to the standalone BiLSTM mismatched DPD reveals that the proposed DPD consistently outperforms the standalone BiLSTM DPD. Figure 9 depicts the spectra measured at the output of linearized DUT with the proposed matched and the proposed mismatched DPD for the case of four different test signals with bandwidths of 20 MHz, 30 MHz, and 40 MHz. These spectra confirm the resilience of the proposed DPD as seen through the ACLR results in Table 4. The results in Table 4 and Figure 9 show that the proposed LUT-assisted BiLSTM DPD enhances the performance of standalone BiLSTM DPD while maintaining its resilience to some of the signal characteristics.

The proposed LUT-assisted BiLSTM digital predistorter was successfully evaluated and compared to the standalone BiLSTM predistorter. The results show that the proposed model has two advantages. First, it outperforms the standalone BiLSTM model by enhancing the ACLR of the linearized amplifier by a few dBs even with a relatively low number of LSTM cells. Furthermore, it was shown that the use of the two-box structure does not hinder the ability of the proposed predistorter to be resilient to change in the signal bandwidth and maintain bandwidth agnostic linearization performance.

## 5. Conclusions

In this paper, a LUT-assisted BiLSTM neural network DPD structure was proposed for the linearization of power amplifiers. The proposed DPD was experimentally validated using 5G NR test signals. It was shown that, compared to the standalone BiLSTM DPD, the proposed structure allows for approximately 5dB enhancement of the ACLR at the output of the linearized amplifier with reasonably sized neural network sub-function. Furthermore, the proposed structure was shown to maintain the signal’s bandwidth agnostic performances of the BiLSTM DPD. Future work will investigate alternative arrangements of the two-box LUT-assisted BiLSTM neural network DPDs and their effect on the model performance and complexity.

## Figures and Tables

**Table 1:** Characteristics of the test signals.

Signal	Bandwidth	Number of Carriers	Sampling Rate	PAPR
10 MHz (1C)	10 MHz	1	122.8 Msps	10.4 dB
20 MHz (1C)	20 MHz	1	122.8 Msps	10.4 dB
20 MHz (2C)	20 MHz	2	122.8 Msps	10.5 dB
30 MHz (2C)	30 MHz	2	153.6 Msps	10.5 dB
40 MHz (1C)	40 MHz	1	153.6 Msps	10.5 dB
40 MHz (4C)	40 MHz	4	153.6 Msps	10.7 dB

**Table 2:** Performance benchmarking of standalone neural networks based DPDs.

Metric	RVTDNN	CNN	Vanilla LSMT	Stacked LSTM	BiLSTM
ACLR Upper	−40.2	−43.5	−42.4	−42.8	−44.2
ACLR Lower	−40.9	−43.7	−42.6	−43.5	−45.4

**Table 3:** Performance benchmarking of the proposed DPD.

DPD Type	ACLR1 [dBc]		ACLR2 [dBc]	
	Lower	Upper	Lower	Upper
No DPD	−31.9	−31.0	−46.5	−45.7
LUT DPD	−40.6	−38.7	−53.1	−52.0
MP DPD	−51.2	−49.3	−51.2	−51.2
BiLSTM DPD (*N_LSTM_* = 10)	−45.1	−44.6	−49.4	−50.5
BiLSTM DPD (*N_LSTM_* = 150)	−45.8	−44.8	−49.8	−50.7
Proposed DPD (*N_LSTM_* = 10)	−49.5	−50.0	−50.8	−51.8

**Table 4:** Performance resilience of the proposed DPD.

Signal	DPD	ACLR1 [dBc]		ACLR2 [dBc]	
		Lower	Upper	Lower	Upper
10 MHz (1C)	Proposed Matched	−51.6	−50.3	−57.7	−57.8
	Proposed Mismatched	−53.8	−53.8	−56.5	−56.7
	BiLSTM Mismatched	−43.1	−43.6	−51.6	−52.1
20 MHz (1C)	Proposed Matched	−50.7	−50.9	−55.3	−56.2
	Proposed Mismatched	−51.9	−52.3	−53.2	−54.0
	BiLSTM Mismatched	−41.9	−42.4	−50.0	−50.7
20 MHz (2C)	Proposed Matched	−50.7	−51.1	−54.8	−55.3
	Proposed Mismatched	−52.4	−52.6	−54.0	−54.6
	BiLSTM Mismatched	−42.5	−43.1	−50.1	−50.8
30 MHz (2C)	Proposed Matched	−50.2	−49.1	−51.9	−52.7
	Proposed Mismatched	−50.9	−51.0	−51.1	−52.5
	BiLSTM Mismatched	−42.6	−43.4	−49.1	−50.5
40 MHz (1C)	Proposed Matched	−48.3	−48.8	−50.3	−51.5
	Proposed Mismatched	−49.1	−49.3	−50.3	−51.5
	BiLSTM Mismatched	−41.3	−42.1	−49.3	−50.5

## Data Availability

The datasets presented in this article are not readily available. Requests to access the datasets should be directed to the corresponding author.
